# Converging Architectures: Precision Biomanufacturing and Soft Robotics Rewiring Tissue Engineering

**DOI:** 10.3390/mi16091052

**Published:** 2025-09-15

**Authors:** Miriam Filippi

**Affiliations:** Soft Robotics Laboratory, ETH Zurich, Tannenstrasse 3, 8092 Zurich, Switzerland; miriam.filippi@srl.ethz.ch

Biomedicine is moving from sculpting tissues to engineering systems. In the last decade, advances in micro- and nano-architectures, three-dimensional (3D) bioprinting, miniaturized machines, soft technologies, and machine-learning-driven design have converged to redefine both diagnostics and therapeutics. The musculoskeletal, cardiovascular, and nervous systems are now being reconstructed in vitro with unprecedented fidelity, while advances in implantable devices and biohybrid biomedical systems extend this progress beyond the lab, unlocking new avenues for therapeutic testing, validation, and ultimately clinical translation. This editorial connects recent work across these domains, emphasizing how structural design, functional integration, and translational validation are converging into a new paradigm of regenerative medicine and personalized healthcare. Moreover, here we argue that future progress will hinge on standardization, integration, and validation, without which translation will stall. If these challenges are met, biofabricated systems will advance from benchtop ingenuity to clinical reality, reshaping diagnostics, therapeutics, and ultimately the very nature of regenerative medicine.

Traditionally, biomaterials were considered passive scaffolds. Today, rather than tailoring scaffolds to fit cells, engineers now tune architecture to instruct tissue function. Impressively, micro- and nanoscale architecture is itself therapeutic [1]. Recent studies on innovative micro- and nano-architectures in biomedical engineering and soft-to-hard biomimetic material tuning demonstrate that pore geometry, stiffness gradients, and nanoscale surface features directly shape cellular behavior, angiogenesis, and immune modulation [2,3,4]. Nanoengineering not only fortifies scaffolds but also directs cells to adopt native-like behaviors [5]. The implication is clear: function follows multiscale form, and controlling architecture becomes as important as selecting cell type or growth factor. Therefore, across soft and hard tissues, micro/nano-architecture is no longer background; it is the intervention.

In addition, thanks to their nanoscale precision and remote controllability, responsive nano- and microsystems endow scaffolds with tunable properties that dynamically steer tissue culture by directly modulating cellular physics. Magnetic nanomaterials, for instance, can reshape cell biomechanics under external fields, influencing processes from polarization and differentiation to apoptosis [6,7]. Similarly, diverse nanomicrosystems can target the membrane potential of bioelectronic cells, such as neurons, enabling neuromodulation with transformative potential for neural implants and biohybrid interfaces [8]. Moreover, cardiac tissues present unique challenges due to the need for electromechanical coupling to guarantee cell functionality. Here, extrinsically conductive nanomaterials emerge as critical enablers, allowing electrical pacing, synchronous contraction, and long-term functionality [9,10].

Whereas micro- and nano-engineering approaches provide the spatial resolution needed to manipulate individual cells and their microenvironments, 3D bioprinting and other biomanufacturing methods extend this control to the mesoscale, enabling the organized assembly of larger tissue architectures that replicate the structural and functional complexity of native organs [11,12,13,14]. To enhance the capabilities of bioprinting and its constructs, machine learning is increasingly employed to optimize processes, select materials, and fine-tune both mechanical and biological performance [15]. Bu navigating the complexity of the biomanufacturing process (arising from the abundance of available materials and strategies), predictive approaches offer the potential for cost-effective solutions [16]. New machine-learning-guided workflows are transforming print quality from artisanal tuning into reproducible, data-driven manufacturing [17].

Specialized biomanufacturing modalities push boundaries in vascularization and neural integration. Vascularized bone bioprinting, chemical and thermo-responsive sacrificial templates and multimaterial extrusion bioprinting of interconnected designs in multicompartment constructs converge on the same goal: perfusable architectures capable of nutrient and oxygen exchange [18,19,20,21,22]. Extrusion-based bioprinting can produce complex architectures useful to recreate the structural intricacy and the heterocellular crosstalk of the nervous systems [23,24]. 3D bioprinting enables platforms for human neural tissues with defined cell types that self-organize into functional neuron–astrocyte networks, providing a powerful tool to study neural circuitry, model disease, and screen therapeutics [25,26]. Laser-based bioprinting stands out for uniting microscale precision with mesoscale tissue assembly, bridging cell-level control and organ-level architecture. For example, laser-assisted printing of capillary-like endothelial patterns and LIST bioprinting of dorsal root ganglion neurons show that even delicate vascular and neural tissues can be patterned with high precision [27,28]. These strategies collectively demonstrate that 3D bioprinting has matured beyond proof-of-concept to a modular platform, where vascular, neural, and structural requirements can be combined within a unified workflow.

Emerging strategies highlight the potential of 3D bioprinting to reconstruct dynamic tissue units, such as engineered contractile muscle tissue. Advances in skeletal muscle bioprinting demonstrate the feasibility of generating tissue replicas with functional maturity, yielding contractile constructs [29,30,31,32,33,34,35]. Looking ahead, integrating these achievements with progress in 3D printing of other components of the musculoskeletal system could enable the full in vitro replication of musculoskeletal embodiment [36,37,38,39]. Stabilizing the interfaces between distinct bioinks is advantageous for heterotypic tissue culture systems, such as engineered muscle–tendon units, that not only execute dynamic functions but also preserve force dissipation and recapitulate the energetic efficiency intrinsic to the native musculoskeletal system [40]. Notably, we are uncovering how specific architectures and material properties can steer cell behavior toward desired outcomes (such as fate specification and differentiation) [5], a consideration that becomes critical for constructs comprising multiple tissue types within a shared culture environment, where fine-tuning media for each tissue is challenging. By leveraging architectural design, we can guide the creation of increasingly biomimetic and high-performance musculoskeletal tissues, ultimately enabling functional dynamic units to study the motion of living beings. Building on this approach, seeding cells onto 3D-printed scaffolds has allowed us to recreate a functional bone–tendon–muscle interface in vitro [41], paving the way for the next frontier: multimaterial bioprinting that seamlessly integrates multiple cell types and tissue regions in a single fabrication step.

With sufficient mesoscale precision, multimaterial bioprinting promises to unlock the creation of fully integrated tissue systems, seamlessly connecting vasculature, connective interfaces, and neural networks, and bringing us closer to replicating the complexity of living organs in vitro. However, the next frontier is not only replicating healthy tissues, but also recapitulating pathologies. In vitro diseased artery models, 3D bioprinted liver disease tissues, tumor microenvironment constructs, and breast tissue models exemplify how architecture and printing converge to create clinically relevant disease platforms [42,43,44,45,46]. These models offer far more than experimental elegance: they provide testbeds for drugs, imaging technologies, and therapeutic delivery systems. By integrating patient-derived cells, such constructs may evolve into predictive tools for personalized therapy selection [47,48].

Credibility in biofabrication depends on rigorous validation when measuring what matters, such as mechanics and fate of our engineered tissue. To this end, mechanical assays are indispensable for capturing the evolving physical properties of living constructs. For example, nanoindentation of soft biological materials provides a direct window into evolving tissue mechanics, ensuring that engineered constructs meet functional requirements [49]. For musculoskeletal system replicas in particular, accurately replicating mechanical properties is critical, as tissue function and force transmission rely on precise biomechanical cues. Complementing these material-level insights, longitudinal monitoring is essential for understanding how constructs behave once implanted. In vivo tracking of tissue-engineered constructs allows researchers to follow graft survival, integration, and remodeling over time [50]. Together, these approaches form the evaluative backbone of translation, ensuring that engineered systems can be trusted as preclinical models and, ultimately, as clinical interventions.

If advanced biomanufacturing gives us the blueprint of life, soft robotics provides the spark, embedding actuation and sensing within the fabrication of living tissues and biomedical devices. Soft robotic micromachines now enable physiomimetic training for engineered tissues, minimally invasive surgical manipulation, and personalized rehabilitation devices [51]. Moreover, over the past two decades, the field of biohybrid soft robotics has advanced toward living machines that merge biological tissues with synthetic components to mimic the complexity and adaptability of natural organisms [52,53,54,55,56,57]. These systems offer advantages such as self-healing, adaptability, and efficient energy use, positioning them for applications ranging from medicine to environmental monitoring. Yet, unlike purely engineered tissues, biohybrid constructs face the added challenge of managing the biotic–abiotic interface. Importantly, as discussed earlier regarding the need for engineered tissues to incorporate physiological distribution networks, the scalability of biohybrids likewise depends on embedded connective fluidic architectures that sustain perfusion, maturation, and functional complexity [58]. Beyond the contribution of advanced tissue engineering, soft robotics adds a crucial dimension by shaping actuators as adaptable, compliant, dynamic components that replicate natural tissue behavior, thereby enhancing the functionality and versatility of biohybrid systems [59]. Moreover, by integrating soft actuators, sensors, and control systems with printed constructs, engineers can reproduce the dynamic microenvironments that drive the development and adaptation of living tissues in the machines [60,61,62,63,64,65]. In this sense, soft robotics does not merely assist biology; it completes it, supplying the feedback loops that render engineered tissues active, responsive, and clinically relevant. This trajectory marks a paradigm shift in medical device design, where soft robotics, guided by biotic-abiotic integration and inspired by the extracellular matrix, promises a new era of autonomous intelligent bio-integrated systems, as well as minimally invasive and biomaterial-driven healthcare solutions.

Taken together, these advances chart a clear trend: architecture defines the microenvironment, bioprinting delivers it with precision, soft robotics incorporates structures for actuation and control, machine learning drives predictive optimization, and rigorous mechanical and imaging validation anchor credibility. With these elements converging, the field is poised to move beyond isolated demonstrations toward integrated, multifunctional constructs (e.g., vascularized bone, innervated muscle, contractile cardiac tissues, and disease-specific organoids) that promise not only to accelerate therapeutic discovery but also to fuel the rise of biohybrid robotics and personalized medicine [66,67].

We call attention to the fact that the convergence of micro/nano-engineering, advanced bioprinting, soft robotics, and machine learning is redefining biomedical engineering, not merely as a branch of materials science, but as a true systems discipline. The emergence of multi-tissue systems that are co-integrated and recapitulate physiological connectivity (including vasculature, innervation, and functional interfaces), and dynamic soft robotic integrations shows us that complexity is no longer a barrier, but a design parameter to be harnessed. In our view, the task ahead is clear: transform manufactured tissues from experimental curiosities into standardized, integrated, and validated systems that will redefine how we diagnose, treat, and ultimately regenerate the human body.
